# Facilitators and barriers to neighborhood social integration

**DOI:** 10.1002/ajcp.70016

**Published:** 2025-09-10

**Authors:** Joelle Fuchs, Deena Shariq, Emily Yang, Giselle Maya, Taylor L. Wilds, Collin W. Mueller, Arianna M. Gard

**Affiliations:** ^1^ Department of Psychology University of Maryland College Park Maryland USA; ^2^ Neuroscience and Cognitive Science Program University of Maryland College Park Maryland USA; ^3^ Department of Psychology The University of Texas at Austin Austin Texas USA; ^4^ Department of Sociology University of Maryland College Park Maryland USA

**Keywords:** adolescence, loneliness, neighborhoods, social cohesion, social integration

## Abstract

Social isolation has reached concerning rates, particularly in the wake of the COVID‐19 pandemic. Social integration is critical to combatting social isolation and loneliness by promoting a sense of community and belonging. Yet, most existing research centers on fostering close personal relationships within family and friend networks. Social integration within the neighborhood context (e.g., chatting with neighbors, participating in local organizations) is another tool that can be used to combat loneliness, but less is known about the process of social integration for residents situated in different sociodemographic groups. The current study examines variability in the process of neighborhood social integration across sociodemographic characteristics (e.g., social role, racial‐ethnic identity, and housing tenure). Thematic analyses were conducted on semi‐structured interviews with 29 residents of Wards 4 and 5 of Washington, D.C. Results suggested that relative to caregivers and community leaders, youth reported fewer opportunities for neighborhood social integration; frequently noted barriers were lack of shared identity, the transient nature of D.C., and school location. Despite sociodemographic heterogeneity in facilitators and barriers to neighborhood social integration, many residents called for more community programming and ‘third places’ to facilitate neighborhood connections.

## Highlights

- Youth reported less social integration into their neighborhoods than other residents.
- Key facilitators and barriers to neighborhood social integration included safety and social cohesion.
- All residents called for community programming and shared spaces for neighborhood social integration.

In 2023, the U.S. Surgeon General released a health advisory describing an epidemic of loneliness and social isolation (Office of the U.S. Surgeon General, 2023), both of which are linked to negative health outcomes, such as internalizing disorders (e.g., anxiety), cognitive impairment, and chronic pain (Umberson & Donnelly, 2023). More Americans than ever are socially disconnected (Kannan & Veazie, 2023), with 22% to 47% of adults in the U.S. experiencing loneliness (Cigna., 2018; DiJulio et al., 2018) and adolescent rates of loneliness skyrocketing since 2010 (Twenge et al., 2019). Reports of social isolation and loneliness increased during the COVID‐19 pandemic as local businesses closed, travel slowed, more people worked from home, and in‐person schooling transitioned to the virtual environment (Holt‐Lunstad, 2022). Although research indicates that neighborhoods can provide social connection to guard against isolation and loneliness (Henry et al., 2014), less is known about how sociodemographic characteristics (e.g., social role; racial‐ethnic identity; housing tenure) interact with the process of neighborhood social integration (i.e., defined as active engagement with the neighborhood context). The present study uses qualitative methods to examine: (1) the places of neighborhood social integration and (2) sociodemographic differences in perceived facilitators and barriers to neighborhood social integration. The goal of this study is to better understand how neighborhoods can be used to support residents' health and well‐being.

## Neighborhood social integration

Socialization theories (e.g., Jencks & Mayer, 1990; Durkheim, 1952) suggest that social integration (i.e., the process through which relationships are created and maintained) enables the development and exchange of social capital (i.e., personal resources, knowledge, support, skills, and value derived from networks; Kawachi & Berkman, 2000; Putnam, 2001), which supports wellbeing (Berkman & Glass, 2000) and minimizes social isolation and loneliness (Holt Lunstad et al., 2010). For example, a young person with limited social integration may seldom interact with their neighbors or attend community events. Missed opportunities to build relationships or contribute to the community reduce their access to social support and community resources (Kim & Ross, 2009), which increases risk for negative outcomes such as cardiovascular disease (Steptoe & Kivimäki, 2013), suicidal ideation (Hatcher & Stubbersfield, 2013), and lower quality of life (Gattino et al., 2013). Conversely, greater social integration, such as interacting with neighbors and participating in local organizations, guards against risk for loneliness (Miao et al., 2019), and fosters greater subjective well‐being (Appau et al., 2019) and a stronger sense of community (Erving & Hills, 2019). Although a body of research links neighborhood social integration to health and well‐being (Holt‐Lunstad et al., 2010) using socialization theories, much of this research relies on static measures of social integration (e.g., social network size, perceived shared values), which prevents an understanding of *how* residents socially integrate into their neighborhoods (Holt‐Lundstad et al., 2010). The present study draws upon theory of “Third Places” (Oldengburg & Brissett, 1982) to better describe the settings of social integration. Additionally, previous work centers experiences of older adults, with limited qualitative research exploring how younger people socially integrate into their neighborhoods (see Child & Lawton, 2019, for a quantitative analysis); thus, the present work elicits narratives from youth in addition to caregivers and community leaders.

## Third places

Some researchers have focused on the power of “Third Places”, or community spaces that are designed to promote social engagement (Oldenburg & Brissett, 1982). Third places like café lounges, parks, and libraries, promote social integration into a space by facilitating social interactions between residents (Eicher & Kawachi, 2011; Small & Adler, 2019). Even small forms of engagement, like talking to a barista (Sandstrom & Dunn, 2014; Schroeder et al., 2022), have been shown to promote neighborhood social integration, community attachment, and group cohesion (McMillan & Chavis, 1986; William & Hipp, 2019; Pendola & Gen, 2008). Changes to third places in a neighborhood can therefore dissolve (Dawkins, 2006; Richardson et al., 2019) or reinforce (Eicher & Kawachi, 2011) existing social support structures and place‐based social ties. Other neighborhood‐level factors, such as crime, may also decrease perceptions of social control and trust among neighbors (Kawachi & Berkman, 2000). This may further impact resident willingness to engage with one another (Bjornstrom et al., 2013; Sampson & Morenoff, 2004). In addition to the role of third places in promoting neighborhood social integration, we must also understand the dual contributions of individual‐ and community‐level factors to the process of social integration.

## The role of individual‐level sociodemographic characteristics in the process of neighborhood social integration

**Figure 1:**
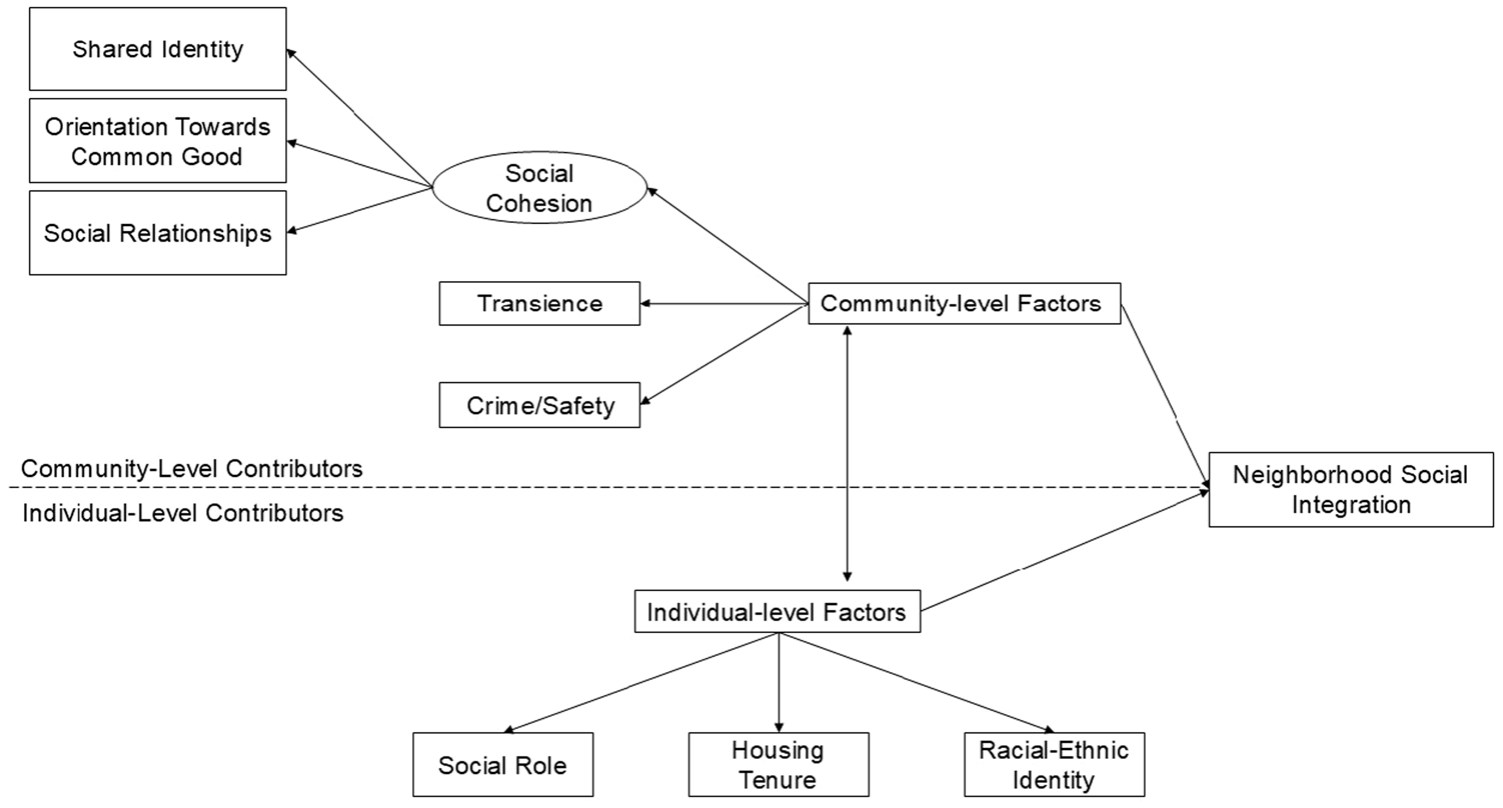
Individual‐ and community‐level contributors to neighborhood social integration. *Note*: Conceptual framing for how individual‐ and community‐level factors contribute to neighborhood social integration closely mirroring Kasarda and Janowitz's (1974) Systemic Model while also feature elements of socialization theories (e.g., Jencks & Mayer, 1990). In this framing, community‐level factors operate as both barriers and facilitators to integration but vary in their relevance/strength by the individual‐level sociodemographic factors of social role (community leaders, caregivers, or youth), housing tenure (residents of <5 years, residents of ≥ 5years), and racial‐ethnic identity (Black or African American, multi‐ethnic, White, Hispanic).

Kasarda and Janowitz's (1974) Systemic Model posits that individual sociodemographic factors (e.g., social position, life cycle stage, length of residence) contribute to the process of neighborhood social integration in conjunction with community‐level factors (e.g., urbanicity, residential stability). For example, economic expansion may lead to the creation or redevelopment of communal places—like libraries or community centers—that promote social integration (Bryson, 2012), or erode long‐standing social relationships through displacement of lower‐resourced residents (Richardson et al., 2019). And yet, such economic changes are not felt equally by all residents: existing research suggests that long‐time residents in hyper‐gentrifying neighborhoods report larger impacts on health, wellbeing, and neighborhood social integration than newer residents (Lim et al., 2017). Variability in the associations between neighborhood social integration and health and wellbeing suggests that residents with differing sociodemographic characteristics (e.g., social role, racial‐ethnic identity, housing tenure) experience community‐level factors differently (Umberson & Donnelly, 2023; Williams & Hipp, 2019). Thus, the Systemic Model compliments framing provided by socialization theories (e.g., Jencks & Mayer, 1990; Durkheim, 1952) and Third Places theory (Oldenburg & Brissett, 1982) because it explicitly defines the transactional associations between individual‐ and community‐level factors in promoting (or inhibiting) neighborhood social integration (see Figure 1).

### Social role

An individual's social role (i.e., youth, caregivers, community leaders) may also be associated with their neighborhood social integration. Adolescence is a period of social reorientation (Crone & Dahl, 2012), during which youth strengthen their social networks and are extrinsically motivated by interactions with peers (Smetana et al., 2006). Youth transition from spending most of their time at homes with family, to more time in their neighborhoods and school settings with peers (Smetana et al., 2006). Peer networks help to buffer against loneliness (Rotenberg et al., 2004) and have been found to mediate the associations between the neighborhood social environment and prosociality (Lenzi et al., 2012), such that greater neighborhood peer support was associated with higher perceived social cohesion and greater prosocial behavior. Caregivers of youth residents, by contrast, engage with the larger neighborhood context, particularly as their children grow older and enter the school system (Bader et al., 2019; Coulton & Spilsbury, 2014). The presence of a child in the household may serve as an impetus for caregivers to engage with health services, join parent‐teacher associations, or volunteer to participate in child‐centered organizations (Hao & Yeung, 2015). Caregivers may use social capital from neighborhood relationships for modeling adaptive parenting behaviors, emotional support, and childcare (Jencks & Mayer, 1990).

Although few studies have directly investigated neighborhood social integration among community leaders (e.g., faith leaders, community organizers/leaders, elected officials), residents with central roles in their neighborhoods have been the subject of health‐intervention research for decades (O'Brien et al., 2009). Given the degree of trust, respect, and understanding that community leaders typically embody in their communities (Purdue, 2001), they may be more inclined to socially integrate to address community issues and promote change (O'Brien et al., 2009). For example, Washington D.C. Advisory Neighborhood Commissioners (ANCs) are locally‐elected officials who represent small neighborhood areas and act as the “voice” of the neighborhood (Office of Advisory Neighborhood Commissions, 2022). ANCs work to bridge the gap between local residents and the D.C. government by advising government agencies on neighborhood issues. Historically, ANCs have provided insight and recommendations on improvement programs, zoning issues, safety concerns, social services, as well as a variety of other neighborhood‐related issues (see Garrison, 2011). A clearer understanding of how community leaders socially integrate into their neighborhoods could reveal mechanisms for effective social change.

### Racial‐ethnic identity

Beyond an individual's social role in their community, racial‐ethnic identity (REI) also influences an individual's experience of neighborhood social integration (Nations et al., 2010; Yi et al., 2016). Race and ethnicity capture sociocultural norms, values, and historical experiences that relate to how individuals navigate the world (Richeson & Sommers, 2016). Shared experiences, structural inequities, and discrimination (e.g., educational and geographical exclusion, legalized disenfranchisement; Pager & Shepherd, 2008) dynamically reinforce cultural and community practices (e.g., faith and religious practices, collectivist orientations, activism) that covary with REI (Barger & Uchino, 2017; Richeson & Sommers, 2016; Yi et al., 2016). A large body of literature has documented culturally‐relevant coping strategies within Black American communities that center social connection through spirituality, community support, activism, and positive racial socialization (Anderson et al., 2018; Jones et al., 2020). In the neighborhood context, these practices may influence resident values and be associated with resident engagement within the neighborhood environment (Nation et al., 2010; Yi et al., 2016).

### Housing tenure

Lastly, housing tenure is associated with the degree of neighborhood social integration (Dawkins, 2006; Oishi & Tsang, 2022). Residential mobility, or the extent of in‐ and out‐migration in a community, may influence residents' ability to create and maintain social ties (Magdol, 2000). Residential mobility has implications for social network expansion, strength of social connections, and perceived loneliness (see review by Oishi & Tsang, 2022). Mobility may erode trust and engagement between residents of differing housing tenures due to lack of shared local experiences (Magdol & Bessel, 2003). Systemic barriers, such as racial segregation and redlining practices have disrupted communities of color through forced residential mobility (Candipan et al., 2021). From 2000 to 2013, Washington D.C. experienced the most gentrification in the United States (by percentage), displacing over 20,000 Black residents (Richardson et al., 2019). Over time, these practices, along with urbanization and economic change disproportionately impact communities of color, highlighting the dynamic interplay between housing tenure and REI in the process of neighborhood social integration.

## Current study

Despite a large body of literature linking neighborhood social integration to health (Bjornstrom et al., 2013; Kawachi & Berkman, 2000; Sampson & Morenoff, 2004; Umberson & Donnelly, 2023; Williams & Hipp, 2019), few studies have qualitatively explored how varied sociodemographic characteristics (i.e., social role, racial‐ethnic identity (REI), and housing tenure) are associated with the *process* of neighborhood social integration, particularly for youth residents (see Browning & Soller, 2014 for a quantitative example). The current study integrates concepts from Socialization Theories (e.g., Jencks & Mayer, 1990; Durkheim, 1952), Third Places (Oldengburg & Brissett, 1982), and the Systemic Model (Kasarda & Janowitz, 1974) to better understand the process of neighborhood social integration. Thematic analyses of qualitative data were used to identify (1) the places of neighborhood social integration and (2) how sociodemographic characteristics dynamically relate to perceptions of barriers and facilitators to neighborhood social integration.

## METHODS

### Participants and procedures

The Community And Resilient Environments (CARE) Project investigates how neighborhood environments shape health and wellbeing across the life course in Washington D.C., USA. The CARE Project engages community stakeholders (i.e., local elected leaders, community organizations, and youth and adult residents) in the research process from project design to data collection and dissemination, drawing on the principles of community‐based participatory research (Wallerstein & Duran, 2010). Analyses for this study draw on 29 semi‐structured interviews (McGrath et al., 2019) collected as primary data.

Washington D.C. presents a unique context to study ecological contributors to community health and wellbeing. Historically dubbed the “Chocolate City” owing to its high concentration of African American residents, thriving Black business base, and broad representation in city government (Asch & Musgrove, 2017), Washington D.C. is home to substantial socioeconomic diversity within Black and African American communities. Documented health disparities by socioeconomic resources and racial‐ethnic identity call upon researchers to work with communities most impacted by environmental inequities and structural oppression (Neblett, 2019). Moreover, recent shifts in racial and socioeconomic demographics (i.e., large losses of Black residents, and an influx of wealthy and highly‐educated White residents; King et al., 2022) that intersect with local narratives of neighborhood change by Black Washingtonians (Prince, 2016; Shinault & Seltzer, 2019) suggest a focus on the nation's capital to advance our understanding of how rising inequality and gentrification impact health and wellbeing across the life course (Schnake‐Mahl et al., 2020).

From Fall 2021 to Spring 2022, 29 semi‐structured qualitative interviews were conducted with residents of Wards 4 and 5 in Washington, D.C. These wards were chosen due to the substantial socioeconomic diversity within majority Black or African American neighborhoods (U.S. Census Bureau, 2023). Participants were purposively recruited from local community organizations including ANCs, faith groups, and public libraries. Eligible participants were 10 years of age or older, spoke English or Amharic, lived or worked within Wards 4 or 5, and consented to audio‐recording. Community leaders self‐identified as individuals holding formal leadership positions in the local community (i.e., faith leaders, community‐based nonprofit workers, ANC representatives). Participants received $40 and University gear for their participation in the study. Informed consent was obtained from all participants (caregiver consent and minor assent for participants <18 years old), and all procedures were approved by the Institutional Review Board of the University of Maryland College Park (UMCP).

### Qualitative interviews

Youth, caregivers, and community leaders participated in 60–90‐min semi‐structured interviews over Zoom. Participants were asked about their perceptions of neighborhood boundaries, neighborhood social ties, youth health and wellbeing, and community outreach and research priorities. All interviews were audio‐recorded, deidentified by research staff, and transcribed using Dedoose®, a data analysis application for qualitative and mixed methods research (Dedoose Version 9.0.17, cloud application for managing, analyzing, and presenting qualitative and mixed method research data, 2021). The current study analyzed participant narratives during the neighborhood social ties section (see Supporting Material for the complete interview guide). During interviews, participants reflected on (a) places in their neighborhood where they and/or their family spend time, (b) people in their neighborhood with whom they spend time and where those interactions occur, (c) whether it is common to see other neighbors interacting with one another, (d) if there are relationships they would like to have in their neighborhood, and (e) whether there are places or resources that would like to have to foster social relationships between neighbors. All referenced names are pseudonyms.

### Demographics

Seven youth (mean age = 13.43 years), 12 caregivers (mean age = 44.08 years), and 10 community leaders (modal age = 45.0 years) participated in the interviews. Forty‐one percent of the sample identified as Black and/or African American, followed by 24% of multiple racial‐ethnic identities, 20% White, 10% Hispanic or Latinx, and 3% Asian. Participants identified as women (44.8%), men (51.7%), or nonbinary (3.4%). Most participants (79%) were residents of Ward 4 and lived in their neighborhood for at least 10 years (55.6%). Within this sample, there were five adolescent‐caregiver dyads or triads, and two caregivers that were also community leaders.

### Positionality statement

All authors acknowledge that their positions influence the construction and interpretation of these findings and share their positions in the spirit of transparency. Below, we've broadly outlined our racial‐ethnic identities, housing residences/tenures, and sociodemographic makeup. These experiences were raised in our discussions of the conceptualization of the neighborhood context and interpretations of resident social dynamics and neighborhood structures. J.F. is a Black cis‐gender woman who grew up in a predominantly White suburban community; she is currently a resident of Maryland. D.S. is a second‐generation Pakistani‐American cis‐gender woman raised in an ethnically‐diverse suburban neighborhood in Maryland; she is a lifelong resident of Maryland. E.Y. is an Asian American cis‐gender woman raised in a predominantly White suburban community in Maryland; she is currently a resident of Northern Virginia. G.M. is a second‐generation Mexican cis‐gender woman who resides in an ethnically diverse suburban community where she was raised. T.W. is a Black cis‐gender woman who grew up in a predominantly White suburban community; she is currently a resident of Texas. C.M. is a cisgender White man who grew up in a middle‐class Houston suburb and now lives in Maryland. A.G. is a White cis‐gender Jewish woman who grew up in both predominantly White suburban communities and sociodemographically‐diverse urban communities in California; she is a resident of Ward 4 in Washington DC. All authors, with the exception of C.M., G.M., and E.Y. took part in data collection; C.M., G.M., and E.Y. joined the team during data analysis.

We are grateful for the community members and local organizations who have shared their time and experiences with our team as part of the CARE Project. Most of our team is Maryland‐based, and we are housed at the University of Maryland College Park. We acknowledge that the university's historical role in denying access and full participation to education has harmed residents in the very communities served by the CARE Project. Although we are committed to rebuilding trust and shared values, we acknowledge that no amount of research or self‐education can replace the lived experiences of any Washingtonian. We welcome all discussions about our positionality as researchers from the University of Maryland College Park.

### Data analytic plan

This study used an abductive coding approach to flexibly explore inductive and deductive themes (Deterding & Waters, 2021). Initial themes (social integration, neighborhood physical environment, safety, transience, and social cohesion) were inductively identified on a subset of interviews between coders (the first author, J.F., and trained undergraduate research assistants, E.Y. and G.M.). No a‐priori conceptual frameworks were used in inductive coding, although all authors were familiar with socialization theories (e.g., Jencks & Mayer, 1990) and past similar research (e.g., Sampson & Morenoff, 2004). Coders then applied deductive coding, guided by the work of Schiefer and Van der Noll (2017), to further define sub‐themes of “social cohesion”. Finally, coders collaboratively created a codebook that included definitions of each theme and Subtheme identified. All transcripts were closed‐coded using Dedoose®, and thematically analyzed to summarize themes within and between sociodemographic characteristics for all residents (Braun & Clarke, 2006).

## RESULTS

### Aim 1: Identify the places of neighborhood social integration

Aim 1 explored the places that residents engaged with in their neighborhoods. Social integration was conceptualized as resident engagement within the neighborhood physical context. Inductive coding of narratives revealed the theme of ***places and activities of engagement (Theme 1)***. This was considered as *what* (e.g., community events and programs), *where* (e.g., parks, libraries, grocery stores), and *how* (e.g., bus, walking, metro), residents used their neighborhoods in their daily routines (Table 1).

**Table 1:** Core themes, definitions, and supporting quotes.

Definitions			Supporting quotes	
***Theme 1. Places and activities of engagement***				
Physical modes of transportation, how they access things they frequently use/what they access within walking distance, and daily routines			“Bars or lounges that are like fairly well‐known that people been patronizing for years, I think these gathering spots, right, for people to um come together and be authentic with themselves but also share a collective identity.”	
Events, programs, happening in the places that they frequently use and access			“[the library] That has all neighborhood events, and that's normally where I find out what, what things are going on, um, and you can get COVID tests from there… information about blood drives…they just have community events posted, and people often use the library as their place for these events.”	
***Theme 2. Multiple dimensions of social cohesion act as facilitators and barriers to neighborhood social integration***				
**Subtheme**		**Definition**		**Supporting quotes**
*Theme 2a. Shared identity*	Similar identities, mindsets, similar goals, expectations			“I mean I would like some more people around my age, just so like I could easily access their house or like hang out with them.”
*Theme 2b. Social relationships*	People who regularly meet and engage with who help individual feel a part of the space			“Postman [is] great. … I've never lived in a place where everybody knows the postman… he's been delivering mail here for 30 years.”
*Theme 2c. Orientation towards common good*	Ideas, thoughts, actions, towards community goals			“It doesn't feel good when you like walk past like the homeless people in the neighborhood, so I, I think some sorta like social services would be nice, 'cause that's exactly what's leading to the situation the neighborhood is nowadays, right now.”
***Theme 3. Safety concerns related to violence and the COVID‐19 pandemic impact social integration***				
Perceived physical threat or support to physical and emotional well‐being			“I wish I had…. places that would feel like a lot safer going to.”	
The COVID‐19 pandemic			“I think the pandemic has made, you know, those interactions a shadow of their former selves…. I think there's remnants of them, you know, and echoes of them, and resurgences of those interactions.”	
***Theme 4. Transient nature of D.C. makes it difficult to form meaningful social connections***				
The fast‐paced energy of neighborhood/D.C.			“DC to me is kinda like, um, New York. People are busy, they have places to go, and they're not, they normally have time to like sit and chat about the small stuff that's going on in life.”	
The frequent coming and going (moving in and out)			“I guess when you own, to like get to know the people around you, and you know, it does feel like a pretty transient, D‐D.C. generally is a transient place, so, um, yeah, but I mean I do think it would be nice to, uh, feel like I knew people, or you know, could like, rely on neighbors and things like that for help and advice.”	

Residents frequently mentioned libraries, parks, recreation centers, and/or grocery stores as places that they and their families patronized. Despite frequent accounts of these places, residents often noted feelings of disconnection and desires to meet others in the community. Thus, social integration was defined by the use and access of physical space and community resources, which is captured by Theme 1. One caregiver compared her current experience to her childhood, indicating a loss of shared spaces for connection:

When I was growing up, …we also all rode the same school bus, right? We all went to the same school, we had the same teachers, …. even if we didn't do the same activities, we started and ended our days in the same place…but here, both parents and kids all get in their cars and leave every day, and go, go someplace else…and then you come in at the end of the day, and you have very little time to do anything, let alone get to know your neighbors…. there's no natural way to like, ‘Hey, I'll see you later at the…’ Or, ‘Hey, would you like to stop at such‐and‐such a place for coffee?’.

When asked about things or relationships that residents would like to have, most mentioned the need for a physical space (e.g., café) or a community event (e.g., block parties, youth programming, and social clubs). A community leader highlighted the unmet potential of a recreation center, noting that “[he] always felt that the recreation center would be a haven for the community to have their social gatherings;” however, he also notes that despite it's potential, “[it's] not happening.” Residents also mentioned a desire for programming. One caregiver mentioned more programming for mothers:

Like maybe if they had like a daytime thing or work at home single mom's that might have an hour or two like maybe between like 12 and 2 like spare before they go one about their day. It could be an exercise class, it could be a wellbeing fitness class, it could just be like a mom's chat class, book‐club type… type of thing you know. Like maybe a book club we can meet somewhere at the library… it would be cool to just be around peers and it would be cool to just you know talk to someone and not feel like it's not meaningful you know?

Additionally, a youth respondent expressed a desire for more inclusive community programming for adolescents:

I would love if uh, the rec center was even more… open … more… accessible…I tried to join the rec team's basketball team, but since there's I believe an age limit of like 13… I would like if they also added one for the teenagers, that would be nice.

Many youth respondents also mentioned home and schools as places that they frequent, though schools were not always located in their neighborhoods. One caregiver directly noted the District's school choice programming has impacted her son; she shared that

“he's inside most of the time…that is a very direct function of going to school outside of the neighborhood.”

### Aim 2: Examine how sociodemographic characteristics interact with the process of neighborhood social integration

Aim 2 evaluated sociodemographic differences in perceptions of facilitators and barriers to neighborhood social integration. Figure 1 provides a conceptual framing of how individual‐ and community‐level factors contributed to neighborhood social integration for interviewed residents. Heavily influenced by Kasarda and Janowitz's (1974) Systemic Model and socialization theories (Jencks & Mayer, 1990), community‐level factors of social cohesion, transience, and crime/safety operated as facilitators and barriers of neighborhood social integration and were generated as themes.

Informed by these models, the following themes were generated from participant voices (Table 1): **Multiple dimensions of social cohesion act as facilitators and barriers to neighborhood social integration (Theme 2); Safety concerns related to crime and the COVID‐19 pandemic impact social integration (Theme 3); Transient nature of D.C. makes it difficult to form meaningful social connections (Theme 4).** Consistent with past literature (Schiefer & Van der Noll, 2017), social cohesion was too broad to capture the nuances of residents' social interactions. Thus, deductive coding was used to create social cohesion sub‐themes of shared identity (Theme 2a), social relationships (Theme 2b), and orientation towards the common good (Theme 2c) to better capture resident experiences (see Table 1).

**Figure 2:**
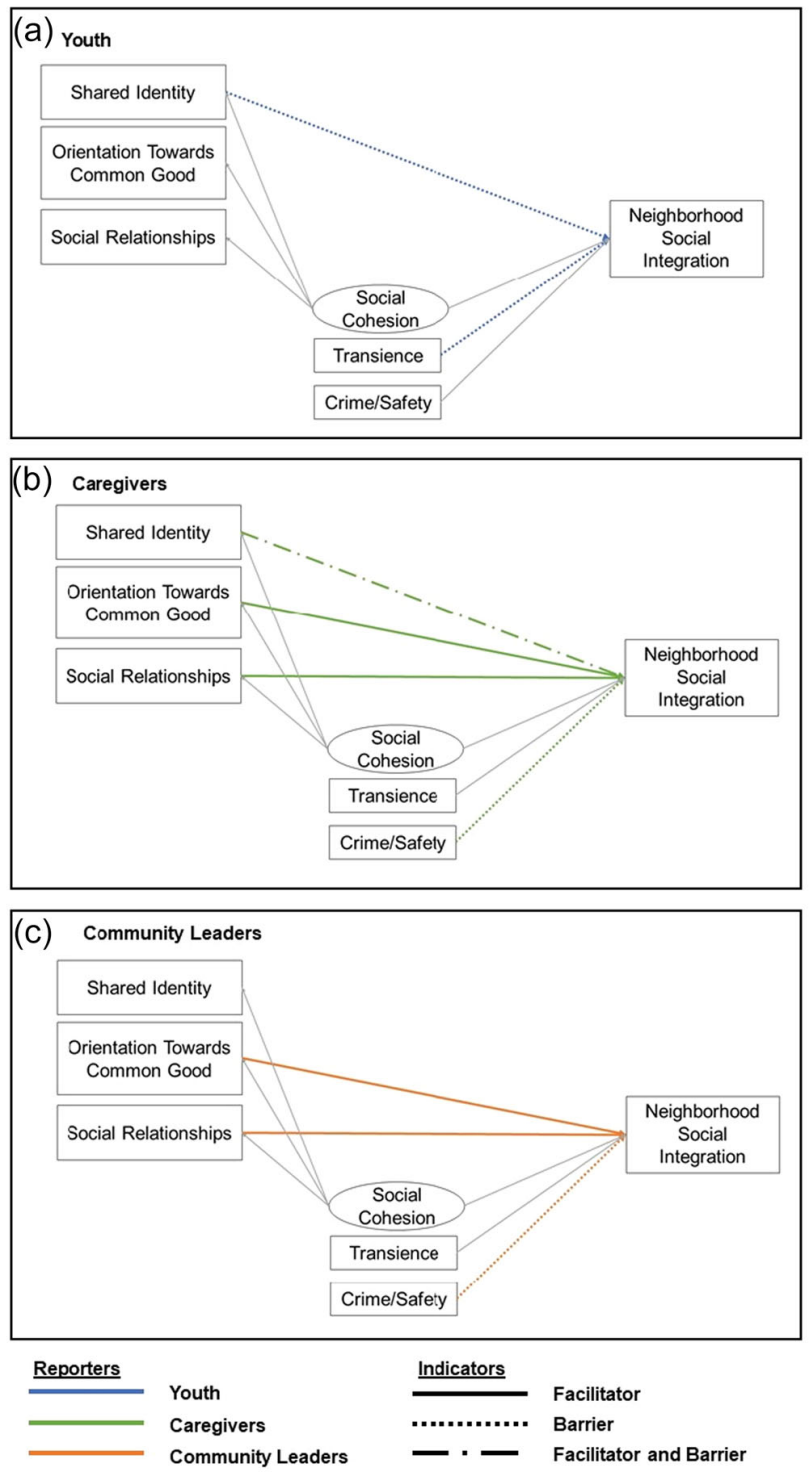
Facilitators and barriers to neighborhood social integration by social role. *Note*: This figure depicts the perceived facilitators and barriers to neighborhood social integration for each social role (youth, caregivers, community leaders). Youth findings are noted in blue (a), caregiver findings in green (b), and community leader findings in orange (c). Barriers are displayed with dotted lines, facilitators with dashed lines, and factors that functioned as both facilitators and barriers are denoted with dotted‐dashed lines.

Associations were found between individual‐level sociodemographic factors and community‐level factors that informed whether they were perceived as facilitators/barriers in the process of neighborhood social integration. Although community leaders, caregivers, and youth reported some similarities in the barriers and facilitators to neighborhood social integration, they also noted differences (see Figure 2). Social role produced the clearest distinctions across themes; thus, results are presented by each role (i.e., community leaders, caregivers, and youth).

Patterns also emerged regarding differences in perceived facilitators and barriers to neighborhood social integration by racial‐ethnic identity and housing tenure. However, most residents who identified as Black, African American, or multi‐ethnic were also residents of over 10 years (86%), while all White identifying residents had only lived in their neighborhood for 4 years or less (50%) or five to 10 years (50%). As our sample size was not large enough to evaluate the intersections of these identities more deeply, results are presented together under the label of Racial‐Ethnic Identity or Housing Tenure.

#### Theme 2: Multiple dimensions of social cohesion act as facilitators and barriers to neighborhood social integration

All residents noted social cohesion as important to their social integration process; however, differences were found in the ways in which aspects were used. Sub‐themes of social cohesion—shared identity, social relationships, and orientation towards the common good—were developed to better capture the nuance of resident experiences.

**Social role**

***Community Leaders***. Leaders often noted the importance of leveraging the social capital of community members, with sub‐themes—orientation towards the common good and social relationships—as the main facilitators of integration, and these two sub‐themes often worked in concert. Leaders reflected on the utility of having various personalities in their neighborhoods and shared how these connections were used to network, gather information, and solve community problems:

You'd be surprised, we have, they're called like block captains…they are people who know everybody on their block and like, when she calls me she's like, ‘Hey Annette, hope you're doing well. I just wanted to talk to you about Ms. Smith up the street. She's having a problem with her handicap parking. Do you know anything about that? And I say yeah, let me talk to Ms. Smith you know … or she'll call me the next day and be like Mr. Jones um, has some young kids up at the high school and they want to do a community clean up. Do know where they can get some … garbage bags and… rakes for the leaves and stuff?’ Those people do exist and they're amazing.

***Caregivers***. Caregivers mentioned all sub‐themes of social cohesion—shared identity, social relationships, and an orientation towards the common good—as facilitators of neighborhood social integration. Social connection with neighbors was fostered by respondents' desires to work together with neighbors to create positive change in the neighborhood (e.g., volunteering with formal mutual aid programs or seeking informal ways to provide social support for elder neighbors). One respondent shared their process of integration as driven by a desire to keep the peace in the area:

I interact with my neighbors ‘cause I live around them and I just want to make sure that I'm keeping it peaceful environment by you know being neighborly cause we live together. We don't know if we gonna need each other. Anything can happen. I look at you every week, so I want to make sure that we're on good terms’.

Shared identity was perceived as both a facilitator of and barrier to neighborhood social integration (Figure 2). When caregivers spoke about the barriers to social integration associated with identity, it was often in the context of not seeing others with similar family structures. A mother noted, “feel[ing] like [they're] like one of the only families with young kids.”

**Racial‐ethnic Identity or Housing Tenure**

Residents who identified as Black or African American often discussed orientation towards the common good and social relationships as facilitators to social integration, while, residents identifying as Hispanic, Latino, or of Spanish origin often mentioned orientation towards the common good.

Residents who lived in their neighborhoods for 4 years or less often perceived orientation towards the common good and social relationships as the facilitators of social integration. Some recent residents leveraged relationships in their apartment complexes as avenues to connect with others. Shared identity functioned as a barrier for many recent residents, as they desired to interact with peers (i.e., single parents, other families). Given that most recent residents were caregivers (83%), these findings may reflect differences by social role, as shared identity was often perceived as a barrier for caregivers in this sample and mentions of identity often reflected their familial status. In contrast, shared identity, in addition to social relationships, was perceived as facilitators for residents who had lived in their neighborhoods for 5 to 10 years.

Um, it seems like a lot of people are new to the area kind of unless they, you know, they've moved…as the young couples … started having kids, so they came from some other place…I mean of course there are older people, I'm thinking of, um, there's a teacher a couple of doors down, and she's probably in her 60's, and, um, I think there are other people sort of, you know, little older in their‐ in their careers, and their lifetime. So, I think it's a mixture, um, but …you know, the really old generation, I don't know of anyone like that so I think it's slowly turning…Our neighborhood was known as a gentrified neighborhood, and we were part of that gentrification… it's just slowly and steadily over the past 12 years… has been people like us who have come with young families and raised their children or so.

#### Theme 3: Safety concerns related to crime and the COVID‐19 pandemic impact social integration

Residents across all social roles mentioned concerns of safety as barriers to social integration, but it came up most often for community leaders and caregivers. Both community leaders and caregivers mentioned concerns of safety as it related to COVID‐19 transmission (Figure 2), whereas concerns regarding crime and loitering were most often brought up by caregivers, White identifying residents, and newer residents (i.e., less than 4 years).

**Social role**

***Community Leaders.*** Many residents reported an inability to use their homes, porches, and stoops as areas for conversations during the COVID‐19 pandemic. However, this was a particularly salient concern for community leaders who emphasized that everyday social interactions were central for their roles in the community, including information‐gathering and community problem‐solving.

***Caregivers.*** Parks, playgrounds, and school events were the spaces that caregivers said fostered social integration; however, interactions in these spaces were hindered by safety concerns regarding the COVID‐19 pandemic. Additionally, caregivers also mentioned safety concerns regarding crime and loitering, which impacted their children's neighborhood. One caregiver shared the ways in which she keeps her children safe in response to not knowing others in the neighborhood:

I grew up in a very small town where…everyone knew each other's name …. you could leave your doors unlocked, and it was just a very safe, open place, very small… now…I don't know everybody in the neighborhood, I have my little small core group of people that I know. My kids are not growing up…with… you know, care‐free safe feel. They know that…when you go outside, mama's gunna lock the door…I guess what I'm trying to say is like, it's definitely not a small‐town feel.

**Racial‐ethnic Identity or Housing Tenure**

Residents who identified as White, Hispanic, Latino, or of Spanish origin predominantly mentioned safety concerns as barriers. Hispanic, Latino, or of Spanish origin identifying residents often mentioned safety within the context of COVID‐19 transmission, whereas White‐identifying residents often discussed safety concerns within both the contexts (e.g., COVID‐19 transmission and crime/loitering, park infrastructure).

Residents who lived in their neighborhoods for 4 years or less often perceived safety concerns as a barrier to social integration, stemming from the COVID‐19 pandemic, in addition to loitering. For residents who lived in their neighborhoods for 5 to 10 years, safety concerns were brought up primarily due to the COVID‐19 pandemic inhibiting travel to community touch points (e.g., grocery store or metro station). One resident mentioned the impact that staying home had on his daily routines:

Being forced to stay home. I mean and part of that is winter. And part of that I think is Omicron, and remote work, you know, just remote work itself keeps you from going out into the neighborhood…Because you're not going to the metro. You're not waiting for the bus…You know. You're not buying lunch out.

#### Theme 4: Transient nature of D.C. makes it difficult to form meaningful social connections

Residents also noted that the fast‐paced energy of D.C., or frequent coming and going, played a role in their ability to social integrate into their neighborhood. Interestingly this theme was often brought up in conjunction with the social cohesion subtheme of shared identity. This was prominent for youth, Black, African American, Multiracial or multi‐ethnic identifying residents, and longer‐term residents (i.e., more than 10 years).

**Social role**

***Youth.*** Although the transience of D.C. was mentioned by all residents, it was especially prevalent for youth. Youth respondents perceived that a lack of same‐aged peers (shared identity), engendered by neighborhood transience, hindered their ability to connect with others in their neighborhoods (Figure 2). Transience hindered social integration through (1) the loss of social relationships that they once had (i.e., friends moving away) and (2) the introduction of younger kids and peers with whom they had no prior relationships with. Youth noted that recreation centers, parks, and school grounds were spaces for connection with others, but that these spaces often lacked peers their age. One teen shared their experience with transience shifting the culture of their neighborhood, and their ability to maintain neighborhood friendships:

…it used to be common but not, necessarily anymore … previously I used to actually know my neighbors by name, but like, now with new people moving in and all, and having their own private lives, I don't necessarily know their names.

Another mentioned that he is unable to see his friends:

So, um, most of my closer friends like, um, Jessie, moved up to Massachusetts…Charles moved over to Maryland… and Josh and Tyrone are in separate wards, so until… our parents communicate with each other, I can't go there either.

**Racial‐ethnic Identity or Housing Tenure**

Transience and shared identity similarly jointly functioned as barriers for residents who identified as Black, African American, and multi‐ethnic or multiracial.

As with endorsements by youth respondents, long‐time residents (i.e., more than 10 years) reported that previously held relationships were jointly impacted by transience and shared identity, in which these factors functioned as barriers.

### Discussion

In this qualitative study with residents in Washington D.C., we sought to identify variability in the process of neighborhood social integration and highlight the perceived facilitators and barriers to this process. Social integration was described by residents as the places and activities that residents engaged with in their neighborhoods. The most common facilitators and/or barriers to neighborhood social integration were the transient nature of D.C., concerns of safety, and multiple aspects of social cohesion—including, orientation towards the common good, shared identity, and social relationships.

#### Limited youth social integration

One of the most glaring conclusions from this study was that youth reported overall fewer opportunities for integration. As social integration has long been studied as a promotive factor for health and wellbeing across the life course (Holt‐Lundstad et al., 2017), these results suggest youth in our sample may not be benefitting from the health‐promoting effects of neighborhood social relationships. A lack of same‐aged peers was a key barrier to neighborhood social integration, fostered by high residential mobility that resulted in long‐time friends moving away and unfamiliar, predominantly younger children moving in. Although much research has linked gentrification to lower perceived social cohesion among older residents (e.g., Versey, 2018), our results suggest that youth may be similarly impacted by the shifts in age and cultural dynamics that parallel renewal and displacement (Richardson et al., 2019). Sadler et al. (2022) found that African American girls living in highly gentrified neighborhoods in Baltimore, MD reported lower neighborhood social cohesion and greater internalizing symptoms. In our sample, the lack of same‐aged peers hindered youth's ability to effectively use their homes to develop neighborhood social networks, which is particularly concerning during a developmental period of heightened social reorientation towards peers (Crone & Dahl, 2012).

Additionally, both youth and caregivers also touched on the power of caregiver monitoring for youth's ability to engage in neighborhood spaces. For youth, most of their time was spent at school or within caregiver‐initiated boundaries (i.e., places with caregivers or at home). Neighborhood uncertainty and danger have long been shown to increase caregiver monitoring (e.g., Jones et al., 2005). Although caregiver monitoring can produce positive benefits for youth living in neighborhoods characterized by high levels of socioeconomic disadvantage and violence (Cuellar et al., 2015), neighborhood cultural changes and transience may perpetuate social isolation through lack of familiarity and trust. Thus, youth are kept closer to home or more spatially restricted than their adult counterparts. Both the Family Stress Model (Masarik & Conger, 2017) and socialization models (e.g., Jencks & Mayer, 1990) suggest that neighborhood social capital allows caregivers opportunities to diffuse caregiving load and stress through local social networks. In this sample, it may be that low social cohesion (i.e., lack of shared identity) urges caregivers to reinforce stricter boundaries for youth due to their inability to leverage community social supports (i.e., peers and neighbors) to ease safety fears. This may explain why caregivers and youth noted frequent use of the home space as a place of social integration for youth, as these are spaces where caregivers are more often present and can oversee the activities of youth.

The last, and perhaps most central, contributing factor to low youth neighborhood social integration was school location. Of the seven teens interviewed, only two attended school within their self‐defined neighborhoods, which is common in D.C. In 2017, over 70 percent of D.C. students attended schools outside of their neighborhoods (Coffin, 2018). These findings highlight youth's limited social integration to primary places of residence (i.e., “First Place”) rather than “Third Places”, or areas that foster informal interactions with others in your neighborhood (Oldenburg, 1997). Despite adolescence being a period where youth are transitioning out of the home and into the neighborhood (Smetana et al., 2006), youth narratives from this study seem to reflect limited integration within their neighborhoods, as they are mainly home (i.e., First Place) or at school (external to their neighborhoods). With limited neighborhood social integration, youth may miss out on the benefits of local community connection (Kim & Ross, 2009).

The limited social integration opportunities for youth also highlight the gap in knowledge of youth narrative on their experiences in their neighborhoods. There is little research on the association between school choice programming and youth neighborhood social integration, with far more work examining impacts on caregivers (Bader et al., 2019). For example, parents of children who go to school inside their neighborhoods perceive their neighborhood boundaries to be smaller and report living in closer‐knit communities (Burdick‐Will, 2018). Although schools critically support the development of youth socioemotional, physical, and cognitive wellbeing (Lipman, 2009), our findings call for more research to investigate the associations between home‐to‐school distance, neighborhood social integration, and youth outcomes from the perspectives of youth themselves.

#### Callsl for more community programming and third places by all residents

Another clear finding from the current study is that neighborhood events are deeply influential, and hold promise for increasing neighborhood social integration. Roughly 45% of residents noted that some form of community programming or gathering would increase connections with their neighbors. Social integration influences health by increasing access to resources and promoting psychological wellbeing through social connection (Kawachi & Berkman, 2000). Block parties, recreation center activities, and volunteering all are examples of community programming that may engage both processes.

There is a large body of literature on the use of community programming for health interventions (Castillo et al., 2019). Community programming can be used to engage community members to advocate for social, structural, and physical environmental equity (Baumann et al., 2021; Chung et al., 2009). Following the 2015 earthquake in Nepal, Baumann and colleagues (2021) described how art (e.g., urban murals, spoken word poetry) was leveraged to promote social cohesion, support, and psychological relief for one community (Baumann et al., 2021). Additionally, intergenerational community programming can further be used to promote social integration across the lifespan. The Learning Families Project was a community‐based intervention designed to emphasize health, happiness, and harmony for residents of all ages (Shen et al., 2017). Programming such as community talks, cooking workshops, craft making, yoga classes, were used to provide opportunities for family members and neighbors to interact with one another and promote neighborhood social cohesion.

Third places are defined by their ability to promote social engagement and enjoyment (Oldenburg & Brissett, 1982). Research has highlighted the importance of third places, such as parks and libraries, as centers vital to social cohesion development (Branas et al., 2018; Oldenburg, 1997; Wan et al., 2021). Many of the community members we interviewed mentioned a lack of shared spaces or age‐appropriate spaces as underlying barriers to forming deep connections with their neighbors. As one youth resident noted, age restrictions in recreations center programming factored in his ability to use the space. More recent work has begun to explore the significance of third places for youth (Littman, 2022; de St Croix & Doherty, 2023; Littman et al., 2024). Work by Littman (2022) and de St Croix and Doherty (2023), for example, highlight the importance of third place research, particularly for socially marginalized youth (e.g., racial‐ethnically minoritized, socioeconomically disadvantaged, etc.) as these places provide opportunities for youth to further develop a sense of belonging and sense of community.

Additionally, a scoping review on of the impact of parks on social cohesion (Wan et al., 2021) proposed that physical characteristics of a space, resident perceptions of safety and accessibility, and usage patterns interact to support the development of social cohesion in a community. Indeed, Branas and colleagues (2018) examined the impacts of a citywide randomized control trial that restored blighted land on resident perceptions violence, fear, and social cohesion. The authors reported that residents living near treated vacant lots reported significantly greater use (by 75%) of outside spaces for relaxing and socializing. Collectively, community programming and third places can foster opportunities for all residents to develop relationships with their neighbors and exchange social capital (e.g., skills, knowledge, tools, perspectives) as mechanisms for promoting health and wellbeing (Holt‐Lunstad et al., 2017; Kawachi & Berkman, 2000), and be particularly important for those that may have more limited access to social spaces outside of the neighborhood.

### Limitations and strengths

Several limitations should be considered alongside the results of this study. Although we observed differences in the perceptions of facilitators and barriers to social integration across racial‐ethnic groups, we lacked saturation to address the nuance of intersectional identities on neighborhood social integration (e.g., Asian *vs.* Hispanic caregivers; new White residents *vs.* longtime Black residents). In our sample, the high correlation between racial‐ethnic identity and housing tenure (i.e., most residents who identified as Black, African American, or multi‐ethnic were residents of their neighborhoods over 10 years, while White‐identifying participants were newer residents to the community) meant that it was difficult to discern the underlying sociodemographic patterns in the process of neighborhood social integration. However, other work has revealed racial‐ethnic differences in social integration patterns, even when controlling for housing tenure (e.g., Nation et al., 2010). Thus, future studies should include a larger sample to assess the nuance of intersectional identities as they relate to social integration.

We also used a sampling approach that was dependent on community partnerships. While this was necessary for engaging neighborhood residents in the study, it limited the generalizability of study findings, as not all resident voices were represented. For example, caregivers in the current study were conceptualized (and thus recruited) as primary caregivers of youth. Second, we acknowledge that the community leaders we interviewed were limited to adults. Future studies must expand the age range of community leaders to gain a deeper understanding of how youth community socially integrate, perhaps through organization and community activism (Ginwright & Cammarota, 2007). Lastly, interviews were conducted with residents from Fall 2021 to Spring 2022, a time when the world was undergoing a series of social transformations. Many of the social behaviors and safety fears that residents noted were in relation to keeping themselves and their children healthy during the COVID‐19 pandemic (e.g., only hanging out at home). As recent research has shown (Ottoni et al., 2022), the COVID‐19 pandemic has forever impacted the ways that individuals and families engage with their communities. It will be important for future research to continue to understand the long‐run impacts of structured social isolation from public health crises on neighborhood social dynamics.

A strength of the study was the addition of youth voice. Youth findings outlined joint and unique experiences of the social integration process. In the current sample, youth predominantly spoke to the transient nature of D.C. and shared identity as perceived barriers to social integration, with connections made to school location and gentrification. More work should explore youth‐reports of neighborhood experiences, as their perspectives are often overlooked or excluded, introducing measurement misalignment and policy implications (Littman, 2022; Pratt et al., 2020). This call also parallels a quantitative research paradigm shift, from using adult‐report to using youth‐report (Lerner et al., 2021; e.g., Browning & Soller, 2014).

### Conclusions and future directions

The current study increases our understanding of the process of neighborhood social integration. Results reiterate the importance of orientation towards the common good, social relationships, and shared identity as key indicators of resident's integration into the neighborhood context. Consistent with socialization theories (e.g., Jencks & Mayer, 1990), neighborhood safety concerns were frequently discussed as a barrier to social integration. At the same time, we found strong associations between individual‐level characteristics and resident experiences of neighborhood social integration; differences in facilitators and barriers emerged in relation to resident social role, racial‐ethnic identity, and housing tenure, with particularly strong differences between youth, caregivers, and community leaders.

For all residents, results highlight the importance of community programming and shared spaces for neighborhood social integration. Place‐based programming may be especially needed in cities like Washington D.C., whose unique historical context and changing population demographics present challenges to neighborhood social integration.

## Supporting information

JF_AJCP_neighborhoodSI_supplemental.
