# Supplementary material for: Facilitators and barriers to neighborhood social integration
Source: Am J Community Psychol. 2025 Sep 10;77(1-2):83–97. doi: 10.1002/ajcp.70016 (PMC13007761; doi:10.1002/ajcp.70016)
Supplement: Supplementary file 1 — JF_AJCP_neighborhoodSI_supplemental. [file AJCP-77-83-s001.docx]

**Supplemental Materials**

**Focus Group / Interview Guide**

**10 – 22 - 2021**

**Version 1.4**

| 1. **Facilitator and Co-Facilitator Name** |  |
| --- | --- |
| 1. **Youth or Parent Focus Group or Interview?** |  |
| 1. **Participant Identification Numbers** |  |
| 1. **Date (mm/dd/yyyy)** | \|___\|___\|/\|___\|___\|/\|___\|___\|___\|___\| |

**Section 1: Information about the study**

***Facilitator Notes:*** *As participants arrive and get comfortable in the room, offer them snacks, and ask them if they have any questions as they fill out the consent form and payment voucher.* *If the focus group takes place over zoom, confirm that everyone has filled out the consent, demographic, and receipt forms through Qualtrics (they will be sent the link ahead of time). If not, you can share the link in the chat feature on Zoom. Once everyone has settled in, all questions have been addressed, and all the paperwork has been completed, read the following script:*

Ok, let’s get started. My name is XXXX. Our lab at Maryland thinks a lot about how environments shape brain and behavioral development in children, teens, and families. The purpose of today is to get an idea of what our neighbors think about their neighborhood. I want to start out by thanking you for coming today on behalf of our research team. We know that your time is valuable, and we appreciate you for sharing your time with us.

I would like to highlight some of the Zoom features you will be using today. Whenever you would like to speak, you can click the microphone icon at the bottom of the Zoom window to unmute yourself. We ask that you mute your audio when you are not speaking. You can also click on the camera icon if you need to stop your video feed at any time.

We value your perspective and ideas. Our research team wants to make sure we remember today’s conversation accurately and do not misrepresent what people say.

So in addition to taking good notes, we will record this meeting so that we can listen to it later. Even though we can see each other on Zoom right now, we are only going to save the audio recording.

You might be wondering what happens to this data after we are done collecting it? We will review all the audiotapes and then our research team will identify common themes and ideas that were raised by lots of people. To report the results of the study, we will prepare a scientific paper and share a report of our findings with community members – including you!

I hope that you will feel welcome to share openly and honestly about your experiences during our conversation today. I want to remind you that we will not disclose your name to anyone else. No one beyond the research team will listen to the audiotapes or know that you participated in today’s conversation. If we quote anything that is said here today in a research report, presentation, or anything else, we will use fake names. The only case in which we would have to break your confidentiality is if, through our conversation, we learn about something that we are required by law to report, like child endangerment.

**[START RECORDING ON ZOOM]**

Ok, let’s get started!

[If there are video quality control issues, turn off video feed of participant and continue with interview. If Zoom connectivity is not working, end the Zoom call. Call participant on their preferred phone number and record phone call on speaker using recorder]

**Section 2: Neighborhood Boundaries and Perceptions**

ICE-BREAKER:

I want to begin with introductions and by asking you a few questions about your neighborhoods. We have invited you here today because you live in Ward 4/5 in NE DC. I’ll invite you to introduce yourself, share what general neighborhood you live in, and how long you have lived here. [PROBE]: Is there something that drew you to live in this neighborhood?

TRANSITION: The focus of our conversation today is to talk about your neighborhood. One thing that comes up when folks talk about their neighborhoods is the question of what is “a neighborhood”. So, what type of things do you think of when you hear the word “neighborhood”?

KEY 1: Now what I would like us to do is think about the boundaries of our neighborhoods.

[Begin screen sharing the Google Maps site and enter participant’s address (the address can be found on the “Cover Sheet”)]. I am showing you a map of your address. Please select “View Option” at the top of your screen and then select “Annotate”. Click “Draw” at the top of your screen and select the “~” option (it will the very first option). Using this tool, draw an outline around what you consider to be your neighborhood. **Ask participant if you need to move the map or zoom in/out.** [Take a screen shot of completed exercise and save]

What were you thinking about when you drew those boundaries? [PROBES]: Did you think about specific places that you and your family go to? **Specific people that you know or hang out with?** Do people in your household work and/or go to school inside of the boundaries you drew around your neighborhood?

TRANSITION: What are you proud of or that you like about your neighborhood? What are things that you would like to see improved?

[PARENTS ONLY] If there was one thing that you would want changed in your neighborhood, what would it be?

KEY 2: Do you feel like there have been efforts to address this issue in your neighborhood?

[PROBES] Who do you think is responsible for addressing this issue? What was the result of that action? How do you feel about the response? Tell me more about that.

**Section 3: Determinants of Social Ties**

INTRODUCTORY: Next, I would like to talk about things that people do in your neighborhood. First, tell me about places that you and your family spend time. PROBE: For example, a library, a church, specific stores, somewhere outside—places that are important to you and your family.

TRANSITION [YOUTH]: What about places you might go without your parents? When you aren’t in school, do you hang out in your neighborhood? What do you do and where do you go? How do you get from place to place?

TRANSITION [PARENTS]: What about your kids specifically? When they aren’t in school, do they hang out in your neighborhood? What do they do and where do they go? How do they get from place to place?

[KEY 1]: Are there people in your neighborhood who you and/or your child interact with on a regular basis (could be daily, weekly, monthly, etc.)? Where do you interact with them and what kinds of things to do you talk about? On the street or sidewalks? Porches? In parks? [PROBES]: How often do you/your child interact with other people in your neighborhood? Tell me more about that.

[KEY 2]: What about people outside of your family? Is it common to see other people talking with each other in your neighborhood? What are they doing? Where are they? On the street or sidewalks? Porches? In parks?

[KEY 3]: Are there things that you would like to do or relationships that you would like to have with people in your neighborhood but don’t? [PROBES]: Do you want to form stronger relationships with other people in your neighborhood – like with neighbors, local officials, a church leader, or business owners? Are there places where you could meet up with neighbors? Places where you would like to meet up, but don’t? What types of places would you like to have in your neighborhood that would allow you to meet up/engage with people in your community/neighborhood? Tell me about that.

**Section 4: Neighborhood Assets and Risks for Child/Adolescent Health**

**YOUTH VERSION**

INTRO: Now we would like to talk about your health and development as a young person. What does it mean for you to be “healthy and happy”? This can include things like diet, physical activity, doing well in school, having friends and hobbies, life goals, etc. [PROBE] What goals do you have for yourself? What do you want to accomplish by the time you end high school?

TRANSITION: For you, what makes it easy or hard to be healthy? This could be things happening in school, in your house, in the world, with your friends…

KEY: Now, thinking about your neighborhood specifically, does your neighborhood make it easy or hard to be “healthy and happy”? [PROBE]: Are there places, people, or things that happen in your neighborhood that make it “healthy” or "unhealthy”? Tell me about that.

**PARENT VERSION**

INTRO: Now we would like to talk about the health and positive development of your kids – specifically young people in your household between 10 and 17 years. What does it mean for your child to be “healthy and happy”? This can include their diet, physical activity, academic performance, friendships, hobbies, etc. Does being healthy mean the same thing as happy? [PROBES] What are your biggest priorities for their development as young people? What does that look like?

TRANSITION: For your child, what makes it easy or hard for them to be healthy? This could be things happening in their school, in the world, with their friends, etc.

TRANSITION/KEY: Now, thinking about your neighborhood specifically, how does your neighborhood support or challenge your child’s health and positive development?

[PROBE]: What are some things in your community that might impact your child’s health, for good or for bad?

Are there places, people, or things that happen in your neighborhood that make it “healthy” or "unhealthy” for your child? Tell me about that. [PROBES]: What about specific places? What about things in your neighborhood that support your child’s development? (if people are only discussing negative aspects/resources and not specific places – we want information on both)

**Section 5: Community Research Interests**

INTRODUCTION: One of our goals is to support teens, families, and communities through outreach, improving access to resources, and by conducting research. But we also know that sometimes community needs don’t match researcher goals. So, I would like to talk about resources and research that you and your family would like to see. For our discussion, when I say “research”, I am referring to participating in research involving humans. There are a few ways you might participate—sometimes you might provide your thoughts in an interview like today, or a survey with lots of questions that you fill out. A research study could happen in person, virtually (phone, zoom), or even through the mail.

TRANSITION: Are there specific questions you have about your/your child’s development? [FOR YOUTH]: Are there specific questions you have about your development as young people? Are there things you want to know about your body and brain?

KEY: I am going to give you a list of potential research topics that our group came up with. Thinking about your/your child’s healthy development, I would like you to rate each of these topics in terms of how important from 1 = not important at all to 5 = very important or most beneficial to your family. Then we can talk about them [SHARE SCREEN – there are two versions, youth and parent].

Research Topics (read them and let people ask questions / provide clarification):

- Social Relationships (peers, neighbors, parents, kids)
- Community Violence
- Environmental Factors (pollution, parks, green space)
- Diet, Physical Activity, and Sleep
- Inequality (income, wealth, school funding)

KEY: Wonderful. Now let’s think about outreach activities and resources. I am now going to show you a list of potential outreach activities that our group came up with. I would like you to rate them from 1 = not important at all to 5 = very important or most beneficial to your family. Then we can talk about them [SHARE SCREEN].

| - Mentoring Activities   - Examples: after school tutoring, paid STEM learning experiences at UMD (e.g., summer internships, tours of research labs) |  |  |
| --- | --- | --- |
| - STEM Career Q&A Sessions   - Examples: Virtual or in-person interactive workshop focusing on “A day in the life of a scientist" - what we do on a day-to-day basis, how do you become a scientist? What types of careers are there for children and teens interested in Science-Technology-Engineering-Math? | | |
| - Stronger Social Media Presence   - Examples: “STEM History Month” highlighting the contributions and careers of diverse scientists | | |
| - Financial Aid Resources and Higher Educations Options   - Examples: Virtual or in-person workshops on the financial aid process and college applications, tours of UMD and other local colleges | |  |
| - “Sharing Science” Events and Information   - Examples: "Science Saturdays" (e.g. presentations and demonstrations in the community, sharing learning resources with families, facilitating museum access) | | |
| - Building Stronger Relationships with Schools   - Examples: reach out to school career counselors to provide additional resources; coordinate career fairs - Hosting / Sponsoring Community Events   - Examples: Attending existing and sponsor new community events (e.g., back-to-school backpack drives, community clean-up) |  |  |

CLOSING: In light of the research and outreach ides we just discussed, do you have any other ideas about how researchers could make a meaningful impact on your family or community? [NOTE** If you run out of time, you can skip reviewing items that were rated low]

**Section 6: Closing**

This conversation has been so helpful. We are so grateful for your offering your ideas and experiences as we seek to learn more about how research can better serve communities in the District. We know that we are still learning, and sometimes we can forget to ask important questions that really matter to people. What did we forget to ask about? What should we keep in mind moving forward? Is there anything else you would like us to know?

Thank you so much for your time and sharing your perspectives and experiences with us.
